# Human–object interaction, connectedness with nature, and life satisfaction: a cross-sectional study

**DOI:** 10.3389/fpsyg.2024.1360518

**Published:** 2024-04-15

**Authors:** Hiroko Kamide, Tatsuo Arai

**Affiliations:** ^1^The Center for Interdisciplinary Studies of Law and Policy (CISLP), Graduate School of Law, Kyoto University, Nagoya, Japan; ^2^Center for Neuroscience and Biomedical Engineering, The University of Electro-Communications, Tokyo, Japan

**Keywords:** human-object interaction, care, learning, connectedness with nature, pro-environmental behavior, life satisfaction

## Abstract

**Introduction:**

This study aimed to elucidate the relationship between interactions with everyday objects (e.g., stationery, clothing, and tools) and one’s connectedness with nature, environmentally conscious behavior, and life satisfaction. While previous research has predominantly explored the link between awareness of and behavior toward nature with direct education and experience related to the natural environment, we focused on the origins of the objects that surround us, which are inherently derived from nature.

**Methods:**

We conducted an online survey with 1,102 Japanese participants, who completed an object–interaction measure assessing the two dimensions of care and learning, and measures of connectedness with nature, pro-environmental behavior, and life satisfaction.

**Results:**

Interactions with everyday objects involving care and learning were significantly associated with a sense of connectedness with nature, pro-environmental behavior, and life satisfaction.

**Discussion:**

The study highlights that interactions with everyday artifacts are not isolated experiences but are related to broader awareness of and behavior toward the natural world, and with individual life satisfaction. Hence, environmental awareness and actions can extend beyond direct environmental experiences and encompass daily interactions with objects. Future research could examine how cultural factors shape the relationship between human–object interactions, connection with nature, environmentally conscious behavior, and life satisfaction.

## 1 Introduction

Research shows that nature has a positive impact on psychological and physical health (Hartig et al., 2003; Wells and Evans, 2003; Bowler et al., 2010; van den Berg et al., 2010; Annerstedt and Währborg, 2011; Lengieza and Swim, 2021). Research has increasingly shed light on the mechanisms by which nature contributes to the overall well-being of mind and body (Ulrich, 1983; Kaplan and Kaplan, 1989; Kaplan, 1995; Rose and Riley, 2023). As our understanding of the psychological aspects of nature has expanded, the concept of connectedness with nature has emerged as a crucial mediating factor in the benefits of exposure to nature (Mayer et al., 2009; Barbiero et al., 2023). Connectedness with nature encompasses emotional bonds such as love and a sense of unity with the natural world (Kals et al., 1999), a sense of belonging to a broader natural community (Mayer and Frantz, 2004), and the integration of nature into one’s self-concept (Schultz, 2001). Given that connectedness with nature is associated with well-being and sustainability, including life satisfaction, environmental concerns, and pro-environmental behaviors (Mayer and Frantz, 2004; Nisbet et al., 2009; Davis and Stroink, 2016; Whitburn et al., 2020; Friedman et al., 2022), extensive research efforts have been dedicated to identifying the factors that foster this connection. These factors include early life experiences with nature (Rosa et al., 2018), controlled experimental exposure to natural settings and green environments (Barton et al., 2016; Richardson et al., 2016), and educational interventions aimed at increasing environmental awareness (Braun and Dierkes, 2017; Lankenau, 2018; Dopko et al., 2019).

Although numerous factors contributing to heightened nature awareness have been studied extensively, a notable gap remains in our understanding of the potential relevance of individuals’ interactions with objects and artifacts in their daily lives to their connectedness with nature. This gap persists because items such as clothing, automobiles, and tools have traditionally been considered symbols of urbanization and industrialization, often perceived as distancing humans from the natural world (Schultz, 2001). Consequently, the relationship between these interactions and individuals’ connectedness with nature has received limited attention.

This study represents the first attempt to shed light on the often overlooked interaction between objects and artifacts, which have traditionally been perceived as disconnected from nature. By introducing the perspective of human-object interaction, this study aims to fill the gap between interaction with objects and their relationship to nature. Specifically, it highlights how mindful interactions with everyday objects may correlate with broader sense of connectedness with nature, pro-environmental behaviors, and individual life satisfaction. Human-object interaction involves two distinct dimensions of everyday interactions with objects. The first dimension pertains to human actions directed toward objects, encompassing elements such as conscientious handling and care of objects. The second dimension involves the learning aspect, wherein individuals develop self-awareness and a heightened appreciation of objects through their interactions with them (Kamide and Arai, 2021). Connectedness with nature refers to the subjective sense of being part of and interconnected with the natural world (Schultz, 2001; Mayer and Frantz, 2004), while pro-environmental behavior entails actively engaging in actions to preserve nature (Brick et al., 2017). Life satisfaction denotes a personal sense of fulfillment in life (Diener et al., 1985). Through this research, a deeper understanding emerges of how interactions with everyday objects may relate to individuals’ relationships with nature, their environmental behaviors, and overall satisfaction with life.

It is crucial to acknowledge that artifacts and objects surrounding individuals are not devoid of connections to the natural realm. Although these items may not immediately evoke thoughts about nature, they are still intricately intertwined with nature through the use of natural materials and complex manufacturing processes. For instance, certain stationery items may contain plastics derived from petroleum, and everyday clothing may be fashioned from natural cotton fibers. Although these objects may appear distant from nature compared to activities such as hiking in a forest or acquiring knowledge about the natural environment, from an alternative perspective, they represent a form of natural derivation. Individuals who engage thoughtfully with these objects may discern subtle traces of nature within them, consequently fostering a sense of connection with the natural world and evoking a sense of contentment through such mindful interactions. Unlike environmental education and forest exploration, which often occur on specific occasions and in specific timeframes, interactions with objects occur daily for most individuals worldwide. Therefore, exploring the potential for nature-related connections through everyday interactions is important.

Myriad objects present in daily life can have multifaceted relationships with human actions and cognition. Ranging from commonplace items such as tables and chairs to contemporary technological gadgets such as smartphones and pens, these objects are typically acquired and utilized for their utilitarian purposes, often leading to people to overlook the depth of their meaning and intrinsic value. Historically, material objects have predominantly been perceived through the lens of capitalist convenience tools (Marx et al., 1948); however, it has since been acknowledged that the objects intertwined with human existence possess not only a straightforward utilitarian significance but also harbor a subjective value that emerges from the complex tapestry of human interactions with them (Mammen, 2017).

In the contemporary milieu, a diverse array of objects populates our global landscape, and there is a growing recognition that the interaction between individuals and these objects constitutes a mutually influential two-way process that significantly affects the human mind. Since materials fundamentally establish behavioral norms for humans, it is imperative to consider not only the influence that humans exert on objects but also the reciprocal influence of objects on humans (Price, 1973). In other words, objects should not be regarded merely as passive entities unilaterally utilized or consumed by individuals; instead, they possess the capacity to exert various effects on people. Hence, it is essential to transcend the surface-level assessment of materials based solely on their utility and durability, and delve deeper into understanding the experiential significance of how objects stimulate human thought processes (Miller, 1987).

The reciprocal relationship between humans and objects has been investigated in diverse contexts. Objects, possessions, and personal belongings play a crucial role in shaping and upholding self-perception and identity, as individuals express, recognize, and affirm their own identities through their possessions (Belk, 1988). Items such as books, paintings, musical instruments, sculptures, and even collections of these objects endow individuals with specific cultural values and social statuses by virtue of their ownership (Bourdieu, 2002).

Moreover, by adopting a more individualistic perspective, cognitive psychology has advanced an approach that delves into the phenomenon of individuals adapting their manner of grasping an object based on their intention to manipulate it. This line of inquiry has been pivotal in elucidating the intricate interplay between cognition and behavior through the study of object manipulation (Rosenbaum et al., 2012). Furthermore, within the realms of computer vision and engineering, technologies have been developed to detect interactions between humans and objects as well as to achieve a comprehensive understanding of various environmental scenes (Gao et al., 2021). Consequently, researchers are currently scrutinizing the interaction between humans and objects in diverse fields and from various perspectives.

Within the framework of object interaction, a central focus of this study, intriguing insights have emerged from the realm of craftsmanship culture (Mori and Kamide, 2018; Mori Masahiro Research Group, 2020). The saying “The act of creating things is the act of creating people” has gained prominence within the context of robot contests. This saying encapsulates the idea that participants in such competitions, through their deep engagement with tools, robots, and other artificial entities, naturally develop a disposition to handle objects meticulously. Consequently, they acquire the ability to cultivate a sense of gratitude toward not only objects but also nature and their fellow beings in the surrounding environment (Mori, 2009).

Similarly, within the context of a traditional tea ceremony, individuals engage with various objects, such as ladles and tea bowls. Through these interactions with objects, individuals embark on a journey of self-improvement, acquiring the capacity to refine minor gestures, social conduct, and interpersonal relationships (Mori, 1991). Furthermore, in the realm of calligraphy, using a brush with a soft tip and correct posture not only serves the practical purpose of conveying thoughts but also exerts a positive influence on cognitive functions such as attention and memory (Kao, 1992a,b). In addition to disciplines like tea ceremony and calligraphy, martial arts such as judo and archery incorporate the concept of “道” (dō), signifying a form of sophisticated artistry transcending mere functionality. Building on this notion, Mori (2023) emphasizes the importance of engaging with objects and technologies not merely for their utility but also with the aim of human development. As observed throughout history, objects have often been treated merely as instruments of utility or monetary value. However, just as individuals gain insights through the care of objects (Kamide and Arai, 2021), there lies the potential for objects to influence and even foster personal growth through interaction with humans.

We investigate how interaction with commonplace objects creates a proclivity for environmental stewardship, a heightened sense of connection with the natural world, and an enhanced personal sense of well-being. Our investigation, drawing inspiration from observations made during a robot contest (Mori, 2009, 2023), shows that genuine interactions with artifacts serve as a catalyst for participants’ contemplation of these objects. While the concept of mutual interplay between objects and one’s self-concept may initially appear unconventional, it becomes more plausible when one recognizes that contact with non-human entities such as nature inherently fosters a profound sense of connectedness with nature (Barton et al., 2016; Richardson et al., 2016; Brambilla et al., 2022; Calogiuri et al., 2023). Consequently, we posit that everyday interactions with objects may be intertwined with broader patterns of pro-environmental behavior and augmented awareness of one’s connectedness with the natural environment.

This study does not purport to establish causality; hence, it refrains from asserting that the aforementioned factors represent the sole mechanism of association. While caring for possessions, attachment driven by factors such as wealth and status may potentially lead to negative psychological consequences. The appreciation of objects as we contemplate interactions with them must be emphasized. The literature suggests that learning something new is positively associated with happiness (Diener et al., 2018). Therefore, we hypothesize that gaining awareness through interactions with objects is linked to life satisfaction. Furthermore, research has established that connectedness with nature is positively correlated with individuals’ well-being (Mayer et al., 2009; Howell et al., 2011; Friedman et al., 2022).

On the one hand, the correlates of pro-environmental behavior are often discussed within the context of potential negative impacts on well-being because of the necessity of making short-term sacrifices (Carmi, 2013). Conversely, some reports suggest that engaging in energy-saving behaviors can relate to well-being and positive affect (Chatelain et al., 2018; Bartolo et al., 2023). This is attributed to the fact that environmentally conscious actions, characterized by their inherent positive values, tend to garner favorable evaluations (Prati et al., 2017). Given the complexities surrounding this relationship, the correlation between pro-environmental behavior and life satisfaction is an intricate and exploratory research topic. Additionally, positing a connection between connectedness with nature and pro-environmental behavior is reasonable. Therefore, this study aims to explore intricate interrelationships between the dimensions of interaction with objects (care and learning), pro-environmental behavior, connection with nature, and life satisfaction.

## 2 Materials and methods

### 2.1 Participants

The survey was conducted by an online survey company in Japan. The company sent the survey request to men and women in their 20s to 60s, who read the explanation of the purpose of this study and agreed to participate. A total of 1,102 individuals (mean age 44.84, *SD* = 14.01, 540 males and 562 females) participated. The occupations of the participants were company employee/public employee (*n* = 458), temporary/contract employee (*n* = 63), part-timer (*n* = 148), student (*n* = 53), housewife (*n* = 168), unemployed (*n* = 129), and other (*n* = 83).

### 2.2 Measurements

#### 2.2.1 Identification of an object

Participants were asked to identify one object that they own and use on a daily basis and to select the closest object categories. The object categories were.

(1) Computers and digital devices, (2) clothing and accessories, (3) objects used for transportation (e.g., bicycles), (4) stationery, (5) objects used for cooking, (6) objects used for medical purposes, (7) tools, (8) objects used for cleaning and laundry, (9) others, and (10) objects used for experiments.

#### 2.2.2 Human–object interaction

We adopted eight items with high factor loadings from the scale used to assess human–object interaction with the selected objects (Kamide and Arai, 2021). The scale has a two-factor structure (Care and Learning) with four items each on a 7-point scale, ranging from “completely disagree” (1) to “completely agree” (7). Care items include “I do not handle the object roughly,” “I pay attention to the object so that it does not break,” “I handle the object with care,” and “I tidy up the object neatly after using it.” Learning items include “I nurture my mental strength while interacting with the object,” “Through interaction with the object, I have developed self-control,” “By interacting with the object, I learn to be kind to those around me,” and “I learn he importance of taking care of objects through my experience with the object.” Cronbach’s test showed good sample reliability for both the care (Cronbach’s alpha = 0.83) and learning factors (Cronbach’s alpha = 0.88). Confirmatory factor analysis was conducted on the two-factor model. All items had factor weights greater than 0.55.

The absolute fit of the model was assessed using the root mean square error of approximation (RMSEA), where a cutoff value of 0.05 indicates an excellent fit to the data (Yuan and Bentler, 2000), and the recommended upper limit is below 0.06–0.08 (Schreiber et al., 2006). For the goodness of fit index (GFI) (Kano and Miura, 2020) and the adjusted goodness of fit index (AGFI) (Bagozzi and Yi, 1988), values greater than 0.90 are recommended. The RMSEA value did not meet the criteria, but otherwise, the model fit was generally favorable (GFI = 0.970, AGFI = 0.940, CFI = 0.972, and RMSEA = 0.079). Composite reliability was 0.88 for Care and 0.83 for Learning; the average variance explained for Care and Learning was 0.66 and 0.56, respectively.

#### 2.2.3 Connectedness with nature

To assess connection with nature, we employed the Nature Connection Index (NCI), which is composed of six items measured on a 7-point scale ranging from “completely disagree” (1) to “completely agree” (7). This scale includes the statements “I always find beauty in nature,” “I always treat nature with respect,” “Being in nature makes me very happy,” “Spending time in nature is very important to me,” “I find being in nature really amazing,” and “I feel part of nature.” Cronbach’s test showed good sample reliability (Cronbach’s alpha = 0.89). Confirmatory factor analysis was conducted on the one-factor model. The results showed all items had factor weights greater than 0.55 and mostly a good fit with GFI = 0.978, AGFI = 0.942, CFI = 0.983, except RMSEA = 0.090. Composite reliability was 0.85, and the average variance explained was 0.55.

#### 2.2.4 Pro-environmental behavior

We used the Recurring Pro-environmental Behavior Scale (REBS) (Brick et al., 2017) to measure environment conservation actions. From the items within this scale, we selected five behaviors that are commonly practiced in Japan. Participants were asked to rate the frequency of their engagement in the following actions: “When you are in PUBLIC, how often do you sort trash for recycling?” “When you are in private, how often do you sort trash for recycling?” “How often do you act to conserve water, when showering, cleaning clothes, dishes, watering plants, or other uses?” “When you visit the grocery store, how often do you use reusable bags?” and “How often do you turn your personal electronics off or in low-power mode when not in use?” Items are rated 1 (Never), 2 (Rarely), 3 (Sometimes), 4 (Often), and 5 (Always). Cronbach’s test showed good sample reliability (Cronbach’s alpha = 0.74). Confirmatory factor analysis was conducted on the one-factor model. The results showed all items had factor weights greater than 0.35 and a good fit with GFI = 0.992, AGFI = 0.968, CFI = 0.984, and RMSEA = 0.067. Composite reliability was 0.73, and average variance explained was 0.37.

#### 2.2.5 Life satisfaction

To assess individuals’ levels of life satisfaction, we used Diener et al.’s (1985) widely utilized Satisfaction with Life Scale (SWLS). This scale comprises the following five items: “In most ways my life is close to my ideal,” “The conditions of my life are excellent,” “I am satisfied with my life,” “So far I have gotten the important things I want in life,” and “If I could live my life over, I would change almost nothing,” rated on a 7-point response scale (7 = Strongly agree, 6 = Agree, 5 = Slightly agree, 4 = Neither agree nor disagree, 3 = Slightly disagree, 2 = Disagree, 1 = Strongly disagree). Cronbach’s test showed good sample reliability (Cronbach’s alpha = 0.74). Confirmatory factor analysis was conducted on the one-factor model. The results showed all items had factor weights greater than 0.65 and good fit with GFI = 0.992, AGFI = 0.972, CFI = 0.996, and RMSEA = 0.061. Composite reliability was 0.91, and the average variance explained was 0.66.

### 2.3 Ethics statement

The study was approved by the Ethics Committee of the Institute of Innovation for Future Society at Nagoya University (protocol code: 2020–34, date of approval: February 3, 2021). All participants provided written informed consent to participate in this study.

## 3 Results

### 3.1 Selection of object categories

Table 1 illustrates the various object categories identified by participants. Notably, some respondents selected musical instruments and objects related to their hobbies, which prompted the creation of two new relevant categories. Computers and digital devices emerged as frequent choices for both male and female participants in their everyday interactions. The most commonly selected object categories, in descending order, included clothing and accessories, items associated with transportation, and stationery. While capturing interaction patterns in terms of object type is significant, it does not represent the primary focus of this study; thus, we will not examine type-specific influences in the ensuing analysis.

**Table 1:** Object categories and number of people selected.

	1	2	3	4	5	6
Men	268	55	109	46	11	12
Women	190	193	42	39	59	12
Total	458	248	151	85	70	24

	7	8	9	10	11	12
Men	18	7	5	5	0	4
Women	1	12	5	4	5	0
Total	19	19	10	9	5	4

(1) computers and digital devices, (2) clothing and accessories, (3) objects used for transportation (e.g., bicycles), (4) stationery, (5) objects used for cooking, (6) objects used for medical purposes, (7) tools, (8) objects related to hobbies (e.g., dolls), (9) objects used for cleaning and laundry, (10) musical instruments, (11) others, (12) objects used for experiments.

### 3.2 Descriptive statistics and correlations

Table 2 presents the descriptive statistics for each variable, and Table 3 displays the correlations between the variables. According to Table 2, Care and Learning were positively correlated within the context of human–object interaction, suggesting that individuals who treat their immediate surroundings with care tend to derive learning experiences from these interactions. Furthermore, the NCI also had positive correlations with Care and Learning, indicating that everyday interactions with objects in one’s immediate environment are associated with a heightened sense of connection with the broader natural world. The REBS was positively correlated with Care and the NCI, implying that careful handling of nearby objects and a sense of connection with nature appear to be related to pro-environmental behaviors. The positive correlation between the REBS and Learning was relatively weak, suggesting that insights gained through interactions with everyday objects were mildly associated with pro-environmental behaviors. Finally, the SWLS score had weak positive relationships with Learning and the NCI, suggesting that learning experiences derived from everyday objects and one’s sense of connection with nature are closely related to individuals’ general life satisfaction.

**Table 2:** Descriptive statistics.

		Care	Learning	NCI	REBS	SWLS
Men	Mean	5.24	4.07	4.58	3.65	3.62
	*SD*	1.10	1.12	1.10	0.74	1.29
Women	Mean	5.51	4.20	4.65	3.95	3.72
	*SD*	1.11	1.14	1.08	0.66	1.42
Total	Mean	5.38	4.14	4.62	3.80	3.67
	*SD*	1.11	1.13	1.09	0.72	1.36

**Table 3:** Inter-variable correlations.

	Learning	NCI	REBS	SWLS
Care	0.48^**^	0.29^**^	0.37^**^	−0.004
Learning		0.33^**^	0.12^**^	0.11^**^
NCI			0.28^**^	0.18^**^
REBS				0.05

^**^p < 0.01.

### 3.3 Overall relationships among all variables

**Figure 1:**
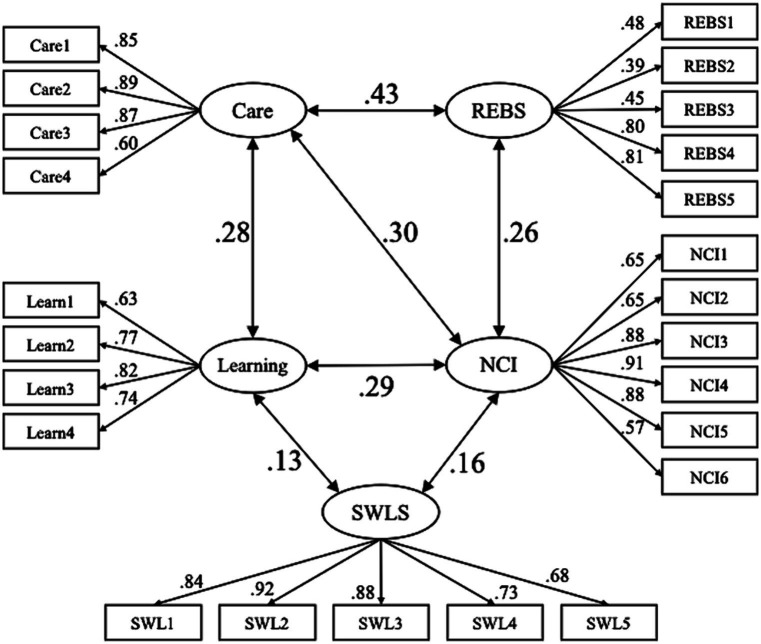
The estimated model illustrating the overall interrelationships among the variables. Circles represent latent variables that were not observed directly, whereas rectangles represent observed measurement variables. The values provided are standardized path coefficients. All paths are significant at *p* < 0.001. Fit indices: GFI = 0.931, AGFI = 0.913, CFI = 0.954, RMSEA = 0.051.

To elucidate the overall interrelationships among all variables, we conducted an analysis using structural equation modeling (SEM). Figure 1 shows the estimation of the final model. Following the correlation analysis, initial paths were established between all latent variables, except for Care and the REBS, as well as the REBS and SWLS, as no significant associations were observed among these pairs. The model was subsequently adjusted based on the significance of the paths and modification indices. The model demonstrated favorable goodness of fit (Figure 1).

The SEM analysis results revealed interrelatedness between the constructs of Care and Learning in interactions with everyday objects, sense of connectedness with nature, pro-environmental behavior, and life satisfaction. The act of cherishing and learning from objects in one’s immediate surroundings is associated with a heightened sense of connectedness with the broader natural environment. Moreover, individuals who handle their immediate objects with care are more likely to engage in environmentally responsible behaviors, such as waste separation and water conservation. Such environmentally considerate actions are linked to an increased awareness of connectedness with nature. Additionally, life satisfaction was associated with experiences of gaining new insights through interactions with everyday objects, concurrently intertwined with connectedness with nature. However, the standardized coefficients were relatively low.

## 4 Discussion

This study aimed to investigate the relationships between interactions with everyday objects, connectedness with nature, pro-environmental behavior, and life satisfaction. While previous research has predominantly focused on factors directly linked to nature, such as environmental education and direct nature experiences, our study drew attention to the fact that everyday artifacts and objects are ultimately derived from nature, and examined their roles in these relationships. We found that interactions with everyday objects such as stationery, clothing, and tools are not isolated experiences but are related to a broader awareness of and behavior toward the natural world behind them. Moreover, these interactions were associated with individual life satisfaction. This discovery underscores the significance of recognizing that environmental awareness and actions can extend beyond direct environmental experiences and encompass daily interactions with objects.

As previously documented, the act of caring for everyday objects is associated with acquisition of knowledge through interactions with these objects (Kamide and Arai, 2021). Individuals can grow through the appreciation of objects not only in terms of specialized activities such as robotics, tea ceremonies, and calligraphy (Mori, 1991, 2009, 2023; Kao, 1992a) but also in everyday interactions with objects in our surroundings. Furthermore, the way individuals engage with objects daily appears to be one of the pathways through which they connect with nature. Perhaps contemplating the origins of objects in front of us and appreciating their significance can promote a more harmonious attitude and behavior toward nature. Additionally, everyday interactions with objects can potentially contribute to an individual’s well-being.

The finding that interactions with non-human objects are related to awareness of one’s connection with nature and environmentally conscious behavior may be influenced by cultural factors. This survey was conducted in Japan for practical reasons, and the Japanese strongly believe that respecting the dignity of objects, acknowledging their inherent life, and coexisting with them (Ekuan, 1994) are crucial. Importantly, rather than anthropomorphizing non-human entities (Epley et al., 2007), non-human objects (e.g., mountains or stones) are believed to inherently possess life and spirituality, which leads to the idea that humans also possess these qualities (Ueno, 2015). By contrast, Western culture tends to view humans as autonomous and rational beings responsible for controlling objects and nature (Coeckelbergh, 2020). This suggests that the results of our study should be approached with caution in terms of generalizability. Future research could explore the significance of interactions with objects from a cultural perspective by comparing such interactions in different cultural contexts.

In this research, the backdrop of the robot contest from which inspiration was drawn is rooted in Buddhist philosophy (Mori, 2023). In the modern context, humans are considered primary, and objects are secondary, perceived as subordinate to humans for their convenience and economic value. However, Buddhism posits that objects and humans exist equally and interconnectedly on the same dimension. The robot contest emphasizes the significance of individuals experiencing growth through immersion in the creative process. A crucial observation is that this sense of growth is absent when one is detached from the external world, focusing on the creation until the boundary between self and the external world fades, leading to a unified state of awareness. This concentration training is referred to as Samadhi in Buddhism and has been discussed in psychology in relation to the concept of flow (Csikszentmihalyi, 1990; Alexander et al., 2021). Considering the human-object interaction involves two aspects: care and learning, it suggests that without a certain level of care with concentration, one may not experience learning, and consequently, may not achieve a connection with nature or happiness. This temporal process and the degree of focused immersion warrant further examination in future studies.

However, there are various limitations to this study. First, it is important to note that this study was cross-sectional, and further investigation is needed to establish causal relationships. External factors not addressed in this study may also play a role. For example, higher socioeconomic status may lead to greater exposure to environmental education, resulting in increased environmental awareness, which could in turn affect other variables. This study represents an initial step in exploring the connection between interactions with objects and nature, focusing on this specific perspective. Future research should consider these alternative possibilities.

Furthermore, while interactions with objects were related to a sense of connectedness with nature and life satisfaction, the coefficients were not particularly high. Although gaining new insights and experiencing a connection with nature are related to individual well-being, life satisfaction and well-being are complex constructs intertwined with a multitude of variables, such as personality (Butkovic et al., 2012; Kandler et al., 2015; Cho et al., 2016; Park et al., 2016), socioeconomic status (Hansen et al., 2008; Singh and Singh, 2008; Chaurasia et al., 2022), and social circumstances (Wood et al., 1989; Zhang et al., 2022). To further investigate the relationship between interactions with objects and well-being from this perspective, a more detailed examination of the underlying mechanisms may be necessary, along with longitudinal studies to explore the duration of these effects.

In this study, we did not specifically compare and investigate the various types of objects people encounter daily because the diversity of these objects is vast. However, it is reasonable to believe the way individuals interact with cherished objects may differ from how they engage with consumable or inexpensive ones. For instance, individuals may meticulously care for their cars while showing less concern for cheap clothing. Additionally, those who cherish expensive items such as musical instruments may treat even disposable items like masks with care. Therefore, it is essential to examine the influence of the type of object and the stability of interactions for individuals.

In addition, participation was confined to individuals who read and consented to the research details online, leading to a potential bias towards those with an interest in the research topic. Furthermore, professionals in specialized fields, such as manufacturing, may exhibit distinct characteristics in their interactions with objects compared to the general workforce. Therefore, future research should consider comparing specific occupations or characteristics to address this potential bias.

In summary, this study provides a new perspective by reevaluating the relationships between interactions with everyday objects, connectedness with nature, pro-environmental behavior, and life satisfaction. As previously highlighted, this study has various limitations and unexplored aspects. However, this serves as a starting point for further investigation, with the aim of advancing our understanding of interactions with objects.

## Data availability statement

The datasets presented in this article are not readily available because the ethical approval conditions do not allow them to be distributed among research teams. The analytical and framework matrices are available upon request from the corresponding author. Requests to access the datasets should be directed to HK, kamide.hiroko.3y@kyoto-u.ac.jp.

## Ethics statement

The studies involving humans were approved by the Ethics Committee of the Institute of Innovation for Future Society at Nagoya University (protocol code: 2020-34, date of approval: February 3, 2021). The studies were conducted in accordance with the local legislation and institutional requirements. The participants provided their written informed consent to participate in this study.

## Author contributions

HK: Conceptualization, Data curation, Formal analysis, Funding acquisition, Investigation, Methodology, Project administration, Writing – original draft, Writing – review & editing. TA: Conceptualization, Supervision, Writing – review & editing.
